# Variations in Gene and Protein Expression in Canine Chondrodystrophic Nucleus Pulposus Cells following Long-Term Three-Dimensional Culture

**DOI:** 10.1371/journal.pone.0063120

**Published:** 2013-05-02

**Authors:** Munetaka Iwata, Hiroki Ochi, Yoshinori Asou, Hirotaka Haro, Takeshi Aikawa, Yasuji Harada, Yoshinori Nezu, Takuya Yogo, Masahiro Tagawa, Yasushi Hara

**Affiliations:** 1 Division of Veterinary Surgery, Department of Veterinary Science, Faculty of Veterinary Medicine, Nippon Veterinary and Life Science University, 1-7-1 Kyonan-cho, Musashino, Tokyo, Japan; 2 Laboratory of Veterinary Microbiology, School of Veterinary Medicine, Faculty of Veterinary Science, Nippon Veterinary and Life Science University, 1-7-1 Kyonancho, Musashino-shi, Tokyo, Japan; 3 Developmental Division of Advanced Orthopedic Therapeutics, Tokyo Medical and Dental University, 1-5-45 Yushima Bunkyo-ku, Tokyo, Japan; 4 Department of Orthopedic Surgery, Graduate School of Medicine and Engineering, University of Yamanashi, Chuo, Yamanashi, Japan; 5 Aikawa Veterinary Medical Center, Tokyo, Japan; Veterinary Surgical Service Japan, 4-3-1 Nishi-ochiai Shinjuku-ku, Tokyo, Japan; National University of Ireland, Ireland

## Abstract

Intervertebral disc (IVD) degeneration greatly affects quality of life. The nucleus pulposus (NP) of chondrodystrophic dog breeds (CDBs) is similar to the human NP, because the cells disappear with age and are replaced by fibrochondrocyte-like cells. However, because IVD develops as early as within the first year of life, we used canines as a model to investigate in vitro the mechanisms underlying IVD degeneration. Specifically, we evaluated the potential of a three-dimensional (3D) culture of healthy NP as an in vitro model system to investigate the mechanisms of IVD degeneration. Agarose hydrogels were populated with healthy NP cells from beagles after performing magnetic resonance imaging, and mRNA expression profiles and pericellular extracellular matrix (ECM) protein distribution were determined. After 25 days of 3D culture, there was a tendency for redifferentiation into the native NP phenotype, and mRNA levels of *Col2A1*, *COMP*, and *CK18* were not significantly different from those of freshly isolated cells. Our findings suggest that long-term 3D culture promoted chondrodystrophic NP redifferentiation through reconstruction of the pericellular microenvironment. Further, lipopolysaccharide (LPS) induced expression of *TNF-α*, *MMP3*, *MMP13*, *VEGF*, and *PGES* mRNA in the 3D cultures, creating a molecular milieu that mimics that of degenerated NP. These results suggest that this in vitro model represents a reliable and cost-effective tool for evaluating new therapies for disc degeneration.

## Introduction

Low back pain resulting from intervertebral disc (IVD) degeneration is a leading cause of incapacity in humans and animals. IVD degeneration leads to loss of proteoglycans and water content in the nucleus pulposus (NP), which contains large amounts of aggregating proteoglycans and type II collagen, typical of compression-resisting tissues [1], [2]. NP cells display a rounded, chondrocyte-like morphology and secrete extracellular matrix (ECM) macromolecules consistent with hyaline cartilage [3]. Cells in the NP originate from the notochord. There is a significant difference in the lifespan of notochordal cells between species, and their loss correlates with early disc degeneration [4], [5]. In pigs, rabbits, rodents, and non-chondrodystrophoid dogs, the notochordal cell population persists into late life [6], [7]. However, in humans, sheep, and chondrodystrophoid breeds (CDBs), such as the Beagle and Dachshund, those cells disappear with age and are replaced by fibrochondrocyte-like cells [4], [8]. CDBs have profound degenerative disc disease with early onset that often develops within the first year [4], [5], [7]. Clinical symptoms derived from abnormal endochondral ossification develop between 3 and 7 years of age, with high incidence and high relative risk of developing disc herniation [7], [9]. Evidence indicates that the chondrodystrophoid phenotype of CDBs is similar to that of humans [10], [11]. Therefore, CDBs are being widely used as a model of human IVD disease. The underlying molecular mechanisms, however, remain poorly understood. *In vitro* cell culture could serve as an important experimental tool, but to our knowledge, no study has examined the phenotype of cultured chondrodystrophic NP cells under different culture conditions. NP cells cultured in monolayers or three-dimensional (3D) scaffolds, such as agarose or alginate hydrogels, exhibit completely different phenotypes depending on the animal species [12]–[15]. For example, porcine NP cells exhibit similar mRNA expression levels in monolayer and alginate cultures, whereas cells in the transition zone are relatively sensitive to culture conditions [15]. By contrast, bovine NP cells exhibit enhanced proteoglycan synthesis in alginate or collagen gels compared with that in monolayers [3]. Although a number of biomaterial scaffolds have been investigated for 3D culture of NP cells, no previous studies have examined the time-dependent alteration of mRNA expression and pericellular ECM compositions of healthy chondrodystrophic NP cells.The objective of this study was to evaluate the phenotype of cultured chondrodystrophic NP cells under different culture conditions. Further, we investigated the potential of 3D-cultured NP cells to mimic the degenerated NP. We hypothesized that long-term culture using agarose hydrogels would mimic the phenotype of *in vivo* chondrodystrophic NP cells, while monolayer culture would promote the fibroblastic phenotype.

## Materials and Methods

### Tissue Acquisition Procedures

Retrieval and use of canine tissue and cells were approved by the Research Ethical Committee at the Nippon Veterinary and Life Science University, Tokyo, Japan and the guardians of the dogs. NP tissue was obtained from 12-month-old male Beagle dogs weighing about 10.0 kg. Euthanasia was induced using pentobarbital sodium (Somnopentyl (50 mg/kg); Kyoritsu Seiyaku Corporation, Tokyo, Japan). Standard lumbar spine magnetic resonance (MR) imaging was performed using a Signa EXCITE 3.0 T (GE Healthcare Japan, Tokyo, Japan) before NP isolation. Healthy NP tissues exhibiting high signal intensities on T2-weighted MR imaging were selected and were classified as grade 1 by the Pfirrmann Grading System [16]. To evaluate phenotypic changes according to Pfirrmann’s grade, we evaluated *type 1 collagen alpha 1* (*Col1A1*), *type II collagen alpha 1* (*Col2A1*) and *Aggrecan* (*ACAN*) mRNA expression in NP tissues (30 discs) classified as grade 1, 2, 3, and herniated NP (HNP).

### Histology and Immunohistochemistry of NP Tissue

Freshly isolated NP tissue samples were classified as described above, after which they were immediately fixed in 4% paraformaldehyde in phosphate-buffered saline (PBS) and then embedded in paraffin. Sections were deparaffinized in xylene, rehydrated through a graded ethanol series, and stained with hematoxylin and eosin (H&E), Safranin-O/fast green, and Von Kossa. For immunodetection of Col1A1, Col2A1, TNF-α, MMP13, and VEGF, the sections were stained with antibodies against Col1A1 (1∶1000, LSL Co., Ltd, Tokyo, Japan), Col2A1 (1∶50, Millipore-Chemicon, Billerica, MA, USA), TNF-α (1∶50, Bioworld Technology, Inc, MN, USA), MMP13 (1∶50, R&D Systems, Inc, MN, USA), VEGF (1∶100, Santa Cruz Biotechnology, Inc., CA, USA), and a biotinylated universal secondary antibody (1∶200, Vector Laboratories, Inc, CA, USA). Sections were incubated overnight at 4°C with primary antibodies, and then secondary antibodies were applied for 20 min at room temperature.

### NP Cell Isolation and Culture

The NP was shredded with scissors and digested in Ham’s F-12 medium (Life Technologies, Carlsbad, CA, USA) containing 1% (v/v) penicillin, streptomycin, nystatin (all antibiotics from Life Technologies), and 0.4% (w/v) pronase (Sigma-Aldrich, St. Louis, MO, USA) at 37°C for 2 hours. The tissue was washed twice with Dulbecco’s modified Eagle’s medium (DMEM)/F-12 and digested in Ham’s F-12 containing 1% (v/v) antibiotics and 0.02% (w/v) collagenase type II (Sigma-Aldrich) for 12 h using the same conditions. The digested tissue was passed through a sterile cell strainer (Falcon, Franklin Lakes, NJ) with a pore size of 100 µm. The filtrate was centrifuged at 2,500 RPM for 5 min to separate the cells from the medium. Cell viability was determined using a trypan blue exclusion test. For 3D agarose cultures, the isolated cells were seeded in 2% low gelling agarose at 5×10^6^ cells/mL. Using a positive displacement pipette, each well of a standard 12-well culture plate was filled with 1.0 mL of agarose and allowed to solidify at 4°C for 20 minutes. Then, the agarose was covered with 1.0 mL of cell–agarose suspension and again allowed to solidify at 4°C for 20 minutes. The cell–agarose layer in each well was covered with 2 mL DMEM/F-12 supplemented with 10% fetal bovine serum (FBS; Life Technologies) and 1% (v/v) antibiotic/antimycotic, and incubated at 37°C in an atmosphere of 5% CO_2_. Culture medium was changed every other day. For monolayer cultures, cells were seeded directly into the wells of a standard 12-well culture plate at a density of 4×10^4^ cells per well. The cultured cells in the monolayer were analyzed with histology, cell proliferation assay, quantification of glycosaminoglycan (sGAG), and mRNA expression studies.

### Cell Proliferation Assay

The proliferation of cultured cells was evaluated using the WST-1 cell proliferation assay (Roche Diagnostics K.K., Tokyo, Japan). Cells were grown for 5, 10, and 25 days in 96-well plates. WST-1 solution was added to each of the wells, and the optical density at 440 nm was determined 1 h later (Powerscan HT; Dainippon Pharmaceutical, Osaka, Japan).

### Histology and Immunohistochemistry of 3D and Monolayer Cultures of NP Cells

For cryosection preparation, tissue samples were immersed in embedding solution (4%CMC; Leica Microsystems) and snap-frozen in liquid nitrogen. Cryosections 10-µm thick were prepared and transferred to SuperFrost slides (Matsunami Glass Industries, Ltd., Osaka, Japan). The sections were stained with H&E for general cell identification. Safranin-O/fast green staining with iron-hematoxylin counterstaining was used to detect secreted pericellular sulfated sGAG, and toluidine blue (pH 2.5 and pH 7.0) was used to detect secreted hyaluronic acid using Ohno’s method [17]. Sections were stained with antibodies against type II collagen (1∶50, Millipore-Chemicon, Billerica, MA, USA), TNF-α (1∶50, Bioworld Technology, Inc, MN, USA), MMP13 (1∶50, R&D Systems, Inc, MN, USA), VEGF (1∶100, Santa Cruz Biotechnology, Inc., CA, USA), Alexa Fluor 488-labeled secondary antibodies (1∶500, Life Technologies) and a biotinylated universal secondary antibody (1∶200, Vector Laboratories, Inc, CA, USA). Sections were incubated overnight at 4°C with primary antibodies, and then secondary antibodies were applied for 20 min at room temperature.

### Quantification of sGAG

Production of sGAG was quantified using the Alcian blue dye-binding assay [18], [19] (Wieslab sGAG Quantitative Kit, Eurodiagnostica, Sweden). Protein samples (extracted using guanidine hydrochloride) were reacted with Alcian blue for 15 min and then spectrophotometrically analyzed at 600 nm using a multidetection microplate reader (Powerscan HT; Dainippon Pharmaceutical, Osaka, Japan). Total sGAG was determined by comparing absorbance values to standard curves of cartilage extract isolated from shark cartilage (Chondroitin sulfate sodium salt from shark cartilage, C4384, Sigma, St. Louis, MO, USA) [18].

### mRNA Expression Studies

At days 0, 5, 10, and 25, total RNA was isolated from cell–agarose and monolayer cultures using TRIzol and quantified by comparing optical densities at 260/280 nm. One microgram of total RNA was reverse-transcribed (Super Script VILO cDNA Synthesis Kit; Invitrogen, Carlsbad, CA) and used to determine the expression of *type I collagen* (*Col1A1*), *type II collagen* (*Col2A1*), *aggrecan* (*ACAN*), *cartilage oligomeric matrix protein* (*COMP*), *alpha 2-macroglobulin (A2M), cytokeratin 18* (*CK18*), and *SRY-related HMG-box 5* and *9* (*Sox5, 9*). For graded NP tissue and cells treated with LPS, the expression of *tumor necrosis factor-alpha* (*TNF-α*), *matrix metalloproteinase 3* (*MMP3*), *matrix metalloproteinase 13* (*MMP13*), *vascular endothelial growth factor* (*VEGF*), and *prostaglandin E synthase* (*PGES*) was analyzed.

Dog-specific primers (Sigma-Aldrich) were designed using Primer Express software, version 3.0 (Applied Biosystems) (Table 1). Polymerase chain reaction (PCR) was performed on a Stratagene Mx3000p System (Agilent Technologies Japan, Ltd.) with Kapa Sybr Fast qPCR Kits (Kapa Biosystems, Inc., Boston, USA). The expression of mRNAs was normalized to that of *beta-actin*, and fold differences were calculated using the ΔΔCt method.

**Table 1:** Primer sequences for realtime PCR.

Gene Name	Gene Symbol	Ref. Sequence	Primer
***Type I collagen, alpha1***	*Col1A1*	NM_001003090	Forward: ACA GCC GCT TCA CCT ACA GT
			Reverse: ATA TCC ATG CCG AAT TCC TG
***Type II collagen, alpha1***	*Col2A1*	NM_001006951	Forward: GAAACTCTGCCACCCTGAAT
			Reverse: GCTGCTCCACCAGTTCTTCT
***Aggrecan***	*ACAN*	NM_001113455	Forward: CTATGAGGACGGCTTTCACC
			Reverse: AGACCTCACCCTCCATCTCC
***Cartilage oligomeric matrix protein***	*COMP*	XM_533869	Forward: GCC GAG ACA CGG ATT TGG
			Reverse: CAC GTC CTC TTG CCC TGA GT
***α-2-Macroglobulin***	*A2M*	XM_534893	Forward: ACT TGG CTC ACT GCC TTT GTA CT
			Reverse: GTT GAG CAG AGA CCC GGA ACT
***Cytokeratin 18***	*CK18*	XM_849849	Forward: AAG AAC CAC GAGGAG GAA GTA AAG
			Reverse: CCC GGA TAT CTG CCA TGA TC
***SRY (sex determining region Y)-box 5***	*Sox5*	XM_003433564	Forward: ACC TCT GAT GGC AAA TCA CC
			Reverse: ATT CAC AAC AGC CAC CTT CC
***SRY (sex determining region Y)-box 9***	*Sox9*	NM_001002978	Forward: TCA TGA AGA TGA CCG ACG AG
			Reverse: GTC CAG TCG TAG CCC TTG AG
***Tumor necrosis factor-alpha***	*TNF-α*	NM_001003244.4	Forward: ACC ACA CTC TTC TGC CTG CT
			Reverse: ACC CAT CTG ACG GCA CTA TC
***Interleukin 1***	*IL-1β*	NM_001003301.1	Forward: TGC AGG TGT CCT CTC AGC TA
			Reverse: GAG CCT GGT CTC ATC TCC AG
***Interleukin 6***	*IL-6*	NM_001003301.1	Forward: GGC TAC TGC TTT CCC TAG CC
			Reverse: GAA GAC GAG GAA GTG CAT CTG
***Matrix metallopeptidase 3***	*MMP3*	NM_001002967.1	Forward: ATG GAG ATG CCC ACT TTG AC
			Reverse: GGA GGA ATC AGA GGG AGG TC
***Matrix metallopeptidase 13***	*MMP13*	XM_536598.2	Forward: TTC TGG CTC ATG CTT TTC CT
			Reverse: GGT CCT TGG AGT GGT CAA GA
***Vascular endothelial growth factor A***	*VEGF*	NM_001003175.2	Forward: TTC CTG CAG CAT AGC AAA TG
			Reverse: AAA TGC TTT CTC CGC TCT GA
***Prostaglandin E synthase***	*PGES*	NM_001122854.1	Forward: AGT ATT GCC GGA GTG ACC AG
			Reverse: GCA GGT CTC CTG ATT GAA CC
***Actin, beta***	*ACTB*	NM_001195845.1	Forward: AGG AAG GAA GGC TGG AAG AG
			Reverse: TGC GTG ACA TCA AGG AGA AG

Dog-specific primers were designed using Primer Express software, version 3.0.

### Lipopolysaccharide Treatment

To determine whether 3D-cultured NP cells mimic degenerated NP cells, we stimulated the 3D-cultured NP cells using lipopolysaccharide (LPS). The 3D-cultured cells were treated with defined media supplemented with a single dose of LPS (30 µg/mL) after 25 days of culture. The mRNA levels and immunohistological localization of *Col2A1*, *TNF-α*, *MMP13,* and *VEGF* were evaluated and compared with those of controls.

### Statistical Analysis

Differences in mRNA expression between graded NP tissues were determined using the Tukey-Kramer method (*Col1A1, Col2A1*, *TNF-α*, *IL-6*, *MMP3*, *MMP13*, *VEGF*, and *PEGS*). Differences in mRNA expression between culture conditions (monolayer and agarose hydrogel) were determined using two-way analysis of variance (ANOVA) with the Tukey-Kramer method. For all the other data, the Mann-Whitney test was applied. Statistical significance was defined as p<0.05. Statistical analyses were performed using StatView 5.0 software (Abacus Concepts Inc., Berkeley, CA).

## Results

### Identification and Selection of Healthy (Non-degenerated) NP Tissue

**Figure 1:**
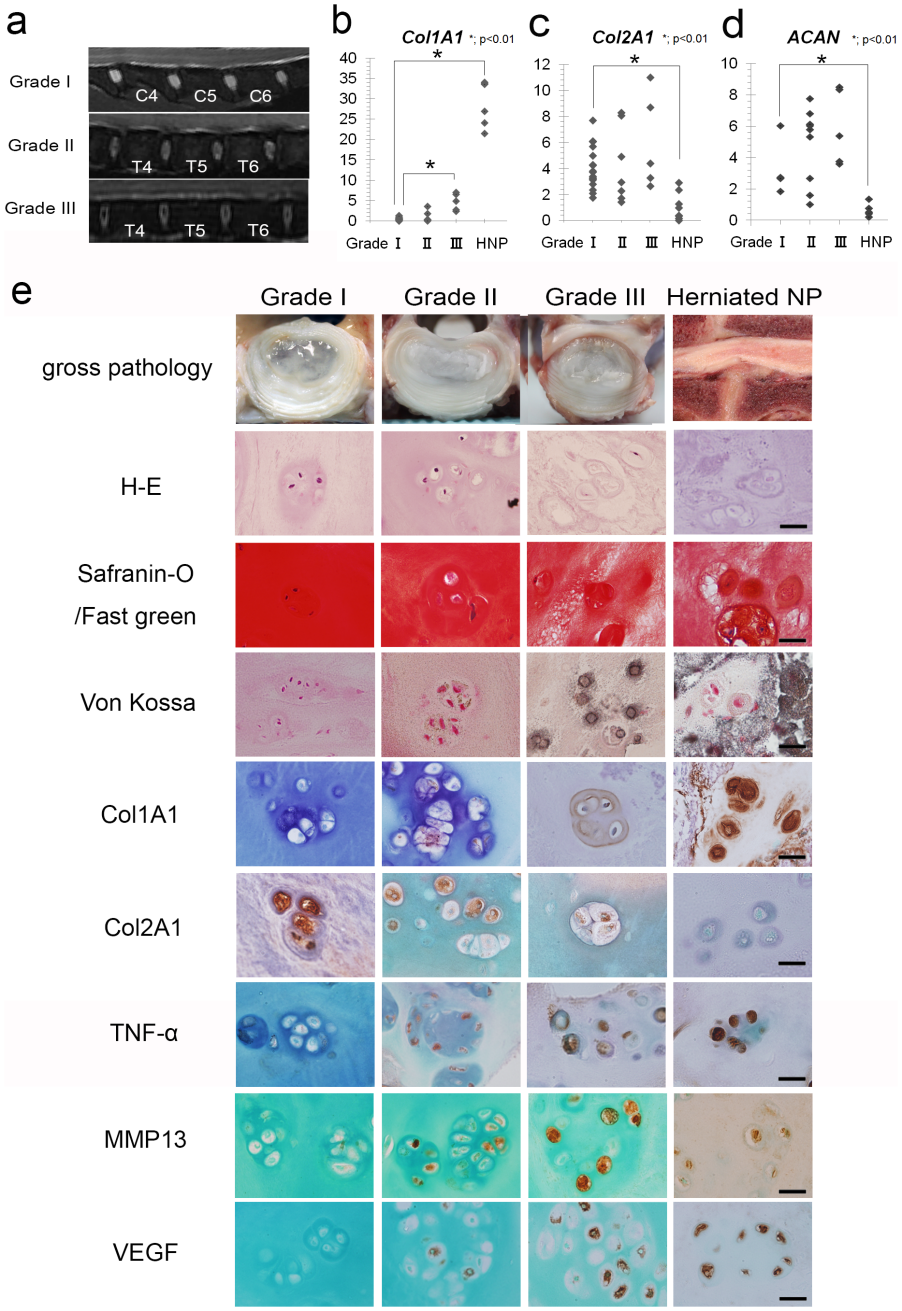
Selection of healthy NP tissue based on MRI. **a)** Healthy NP tissues exhibiting high signal intensity on T2-weighted MR imaging were selected and were classified as grade 1 by the Pfirrmann Grading System. **b–d)** Expression of *Col1A1*, *Col2A1*, and *ACAN* in NP tissues according to Pfirrmann’s grades 1–3 and HNP were analyzed using RT-PCR. Grade 3 NP and HNP tissues exhibited significantly higher expression of *Col1A1* than did grade1 NP tissues. For *Col2A1* and *ACAN*, there was a significant difference only in HNP (Fig. 1c, d), *p<0.05. **d)** Histochemical analysis of sections of NP tissues classified according to Pfirrmann’s grades 1–3 and HNP. Grade3 and HNP cells exhibited typical degenerative histological changes. Scale bar: 20 µm.

To identify healthy NP tissue, we graded the NP tissue specimens based on MR imaging findings according to Pfirrmann’s Grading System [16] (Fig. 1a). We then evaluated the expression of *Col1A1*, *Col2A*, and *ACAN* mRNA in each group. Although all NP tissue was derived from 12-month-old CDBs, grade 3 NP tissues were detected that exhibited significantly higher expression of *Col1A1* compared with grade1 tissues (Fig. 1b). Moreover, 2 of 7 grade 2 NP tissues also exhibited high expression of *Col1A1*. Hence, neither grade 2 nor grade 3 NP tissues were considered suitable for use in experiments because of their differentiated fibroblastic phenotype. For *Col2A1* and *ACAN*, there was a significant difference only in HNP (Fig. 1c and d). According to these observations, we selected NP tissues classified as grade 1 as healthy (non-degenerated) control samples.

### Herniated Canine NP Cells showed Typical Degenerative Histological Changes and Upregulation of Inflammatory and Catabolic Cytokine Levels

**Figure 2:**
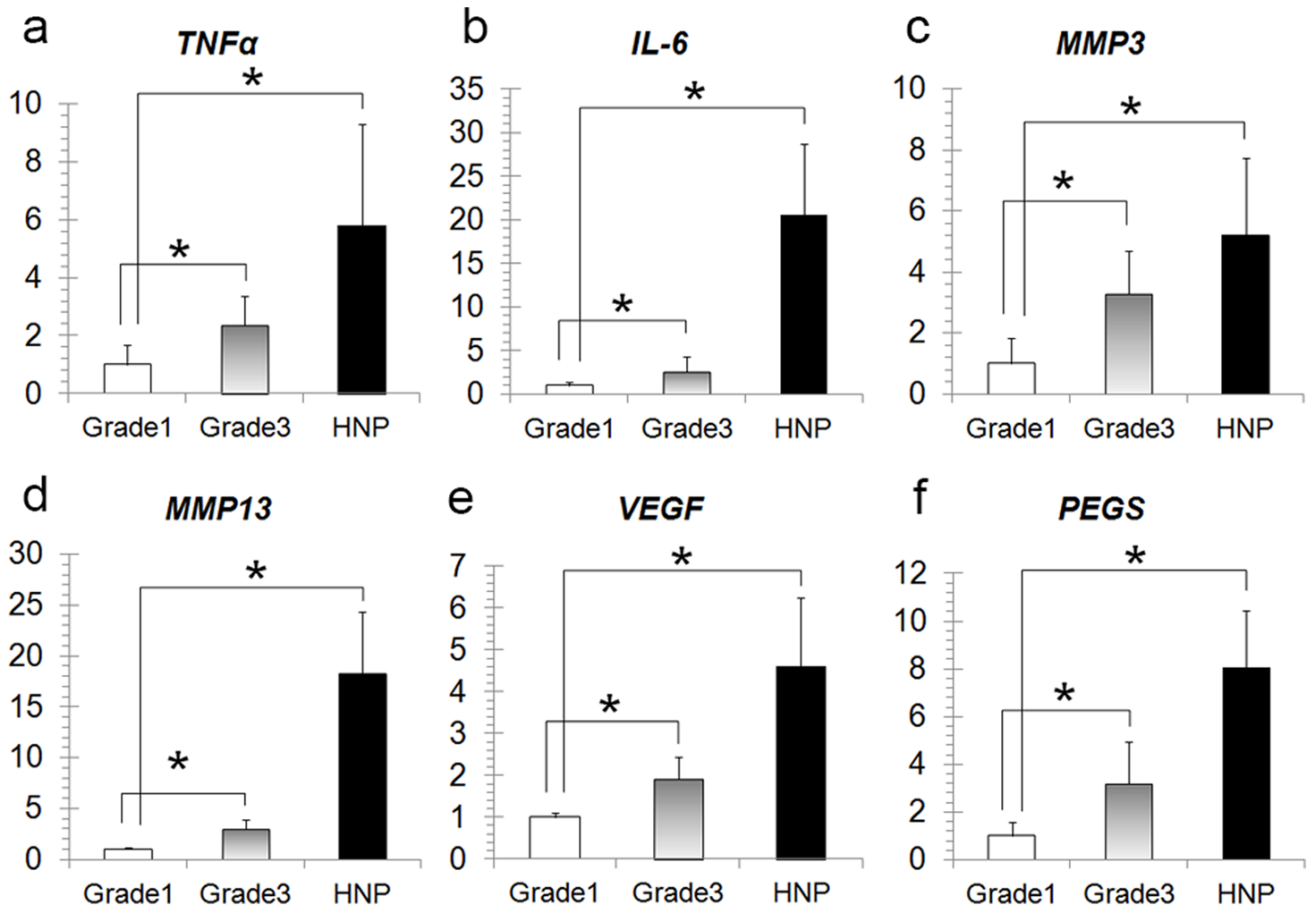
Canine HNP cells showed upregulation of inflammatory and catabolic cytokines. RT-PCR analysis showed high mRNA expression levels of *Col1A1* (Fig. 1b), *TNF-α* (a), IL-6 (b), *MMP3* (c), *MMP13* (d), *VEGF* (e), and *PEGS* (f) in canine HNP cells.

Sections of NP tissues judged as Pfirrmann’s grades 1–3, or HNP showed typical degenerative changes [1], [2] (Fig. 1e). Further, Real-time PCR (RT-PCR) analysis showed high levels of *Col1A1* (Fig. 1b), *TNF-α*, *MMP3*, *MMP13*, *VEGF*, and *PEGS* mRNA expression in canine HNP (Fig. 2a–f).

### Chondrodystrophic NP Cells in 3D Cultures Expressed High Levels of sGAG, Hyaluronic Acid, and Type II Collagen

**Figure 3:**
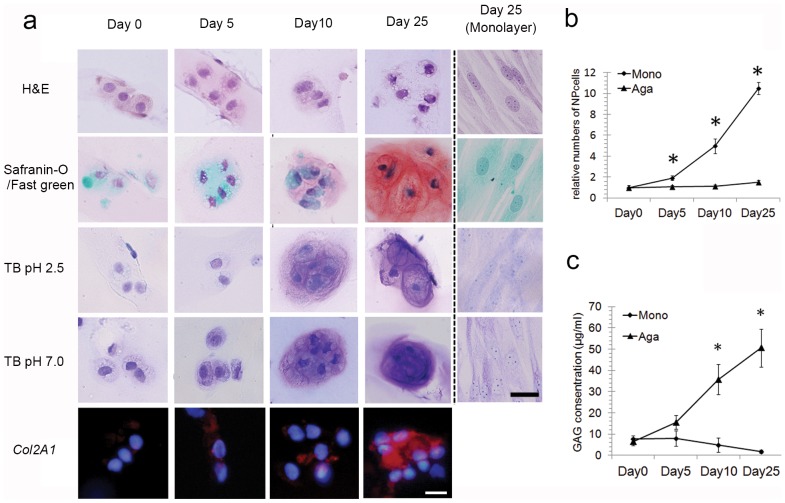
Evaluation of chondrodystrophic NP cells in 3D culture. **a)** Histological characterization of 3D-cultured cells. Chondrodystrophic NP cells encapsulated in agarose hydrogels displayed a rounded and native NP cell morphology and expressed high levels of sGAG, hyaluronic acid, and *Col2A1* in a time-dependent manner, particularly at day 25. In contrast, monolayer cultures at day 25 were negative for sGAG and hyaluronic acid. Scale bar: 20 µm. **b)** NP cell proliferation in monolayers or agarose hydrogels. NP cells did not proliferate when cultured in agarose hydrogel scaffolds. In contrast, in monolayer cultures, the number of cells was 10-fold higher at day 25 than at day 0, *p<0.01. **c)** Quantitation of secreted sGAG using an Alcian blue dye-binding assay. Synthesis of sGAG was significantly higher and increased in a time-dependent manner in agarose 3D cultures of NP cells at day 10 and 25 compared with monolayer cultures (p<0.01), *p<0.01.

At days 10 and 25, chondrodystrophic NP cells encapsulated in agarose hydrogels displayed a rounded and native NP cell morphology. Further, the cells expressed high levels of sGAG and hyaluronic acid in a time-dependent manner, reaching peak levels at day 25 (Fig. 3a). In contrast, 25-day monolayer cultures were negative for sGAG and hyaluronic acid (Fig. 3a). Immunohistochemical analysis revealed the presence of pericellular type II collagen secreted by NP cells cultured in agarose hydrogels; moreover, pericellular type II collagen was found to be strongly positive at day 25 of culture (Fig. 3a).

### NP Cells Failed to Proliferate in 3D Agarose Hydrogels

The number of NP cells grown in agarose hydrogels scaffolds did not increase over the course of the experiment (Fig. 3b). In contrast, cells grown as a monolayer proliferated significantly; at day 25, the number of cells was approximately 10-fold higher than that at day 0.

### The Synthesis of sGAG Synthesis Increased in Long-term Agarose Cultures of Chondrodystrophic NP Cells

Synthesis of sGAG was significantly higher in agarose 3D cultures of NP cells at day 10 and 25 than in monolayer cultures (p<0.01; Fig. 3c). In agarose 3D cultures, synthesis of sGAG increased in a time-dependent manner, while that in monolayer cultures decreased over time (Fig. 3c).

### Upregulation of NP Cell Marker Genes in Long-term 3D Cultures of Chondrodystrophic NP Cells

**Figure 4:**
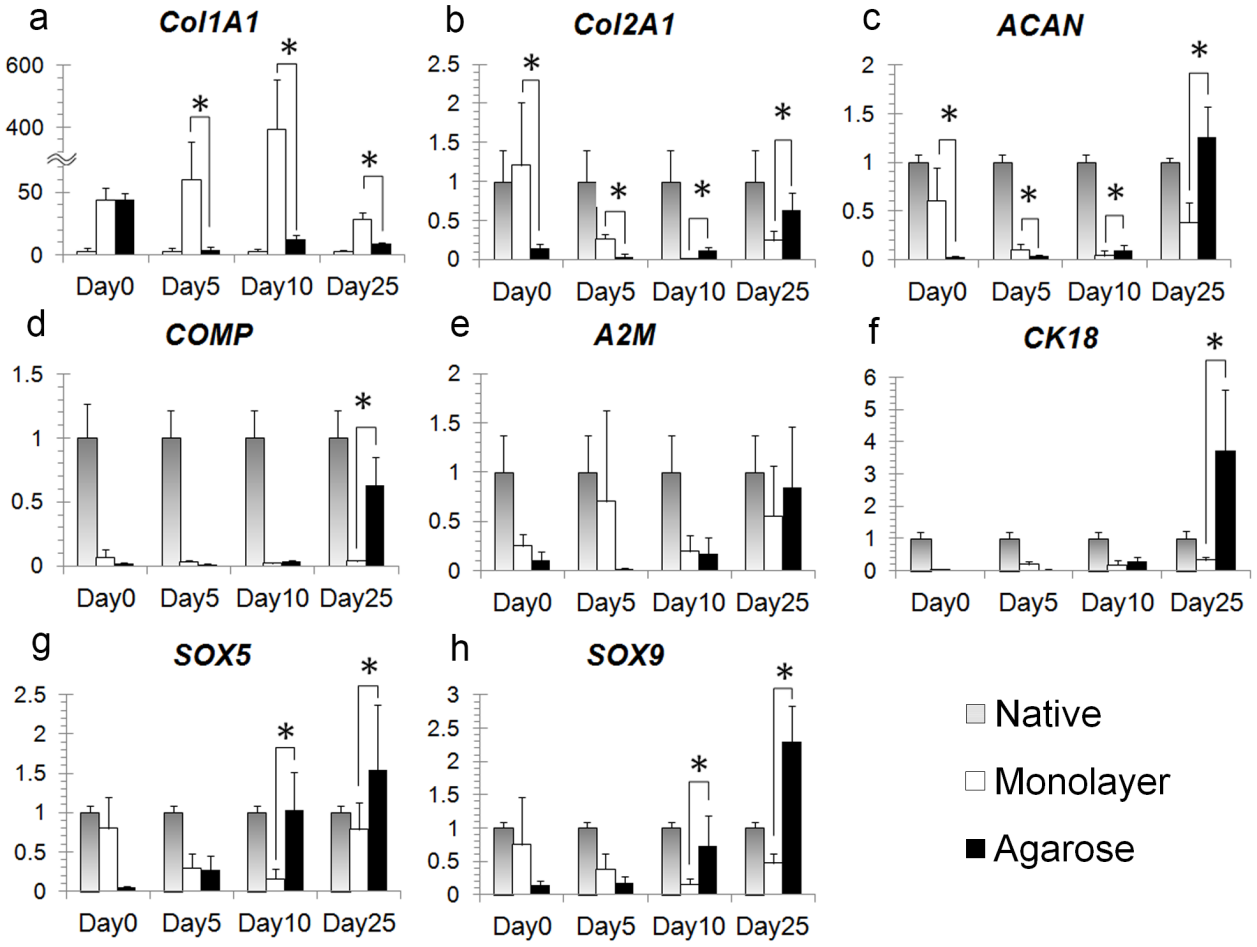
Levels of mRNA expression in chondrodystrophic NP in 3D cultures. **a)** In agarose hydrogels, mRNA expression of *Col1A1* was decreased for all culture periods compared with monolayer culture (p<0.01). **b, c)** *Col2A1* and *ACAN* expression levels were also increased at day 10 and 25 and peaked at day 25 (p<0.01). At early time points (day 0 and 5), agarose cultures exhibited lower expression of *Col2A1* and *ACAN* than monolayer cultures (p<0.01). **d, f)** Expression levels of *COMP* and *CK18* mRNA were increased at day 25 (p<0.01). **e)** In contrast, no statistically significant differences in gene expression were observed in *A2M* expression at day 25. **g, h)** Furthermore, in 3D agarose cultures, NP cells exhibited high expression of *SOX5* and *SOX9* at day 10 and day 25 (p<0.01) compared with monolayers. *p<0.01.

In agarose hydrogels, the expression of *Col1A1* mRNA by cells cultured in agarose hydrogels was decreased in all culture periods compared with monolayer cultures (p<0.01, Fig. 4a). Further, *Col2A1* and *ACAN* expression was increased at days 10 and 25, peaking at day 25 (p<0.01; Fig. 4b and c). At early times (day 0 and 5), agarose cultures exhibited low expression of *Col2A1* and *ACAN* compared with monolayer cultures (p<0.01; Fig. 4b and c). The expression *COMP* mRNA was increased at day 25 (p<0.01, Fig. 4d); however, the difference was not significantly different between 3D and monolayer cultures at days 5 and 10. The expression of*CK18* mRNA was increased at day 25 in 3D agarose cultures compared with monolayers (p<0.01, Fig. 4f). In contrast, no statistically significant differences in mRNA expression were observed in *A2M* expression at day 25 (Fig. 4e). In 3D agarose cultures, NP cells exhibited high expression of *Sox5* and *Sox9* at days 10 (p<0.01) and 25 (p<0.01) compared with monolayer cultures (Fig. 4g and h). The levels of *Col2A1*, *ACAN*, and *COMP* mRNA expression were similar to levels in freshly isolated cells at day 25.

### LPS-induced Inflammatory and Catabolic Cytokine Expression in 3D Cultures of NP Cells

**Figure 5:**
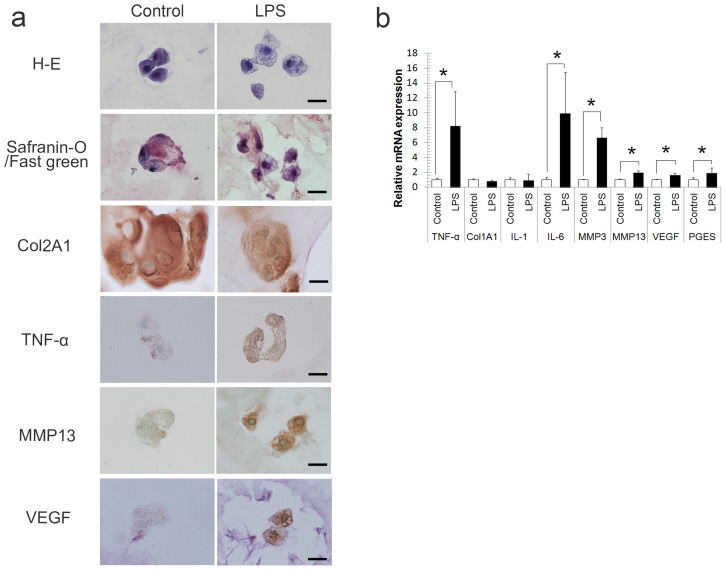
LPS-induced expression of inflammatory and catabolic cytokines in 3D cultured NP cells. Cells were treated with defined media supplemented with a single dose of LPS (30 µg/mL) after 25 days of culture (Fig. 5). After stimulation with LPS, *TNF-α*, *MMP3*, *MMP13*, *VEGF*, and *PEGS* mRNA expression levels were elevated.

To determine whether 3D-cultured NP cells mimicked degenerated NP cells, we stimulated the 3D-cultured NP cells using LPS and evaluated the expression of inflammatory and catabolic cytokines (Fig. 5a and b). LPS treatment activated the expression of *TNF-α*, *IL-6*, *MMP3*, *MMP13*, *VEGF*, and *PEGS* mRNAs (Fig. 5b). Immunohistochemical analysis revealed that *TNF-α*, *MMP13* and *VEGF* synthesis was increased in LPS-treated cells, indicating that LPS induced the expression of inflammatory and catabolic cytokines, thus mimicking the phenotype of degenerated NP cells in 3D culture (Fig. 5a). In addition, *Col1A1* and *Col2A1* protein levels were not affected by LPS treatment.

## Discussion

In the present study, we evaluated the level of expression of mRNA and the composition of the pericellular ECM of healthy chondrodystrophic NP cells cultured in long-term 3D agarose hydrogels that mimic the microenvironment of the native tissue. To our knowledge, this is the first study describing the phenotypic characteristics of cultured chondrodystrophic NP cells under different culture conditions. CDBs are a suitable model to investigate IVD degeneration [10], [11]. Moreover, CDBs suffer from profound degenerative disc disease with early onset, often developing within the first year of life [4], [5], [7]. Therefore, determination and selection of healthy (non-degenerated) NP tissue before any experiment is essential. However, to our knowledge, no report describes the selection of healthy NP tissues derived from CDBs based on MRI. A previous report described that the loss of disc signal on T2-weighted MRI correlates with the progressive degenerative changes of the human intervertebral disc [16]. Further, fibroblast-like cells may replace the chondrocyte-like cells of the nucleus during the degenerative process [2]. The results of our present study show that mRNA expression of *Col1A1* increased as degeneration in NP tissues progressed. By contrast, the protein levels of Col2A1 and ACAN, a marker of NP, decreased as the severity of degeneration increased [19]. However, no significant difference in Col2A1 and ACAN mRNA expression levels was found between grades. This result suggests that reconstruction of the ECM was upregulated in the early stage of degeneration [20]. We therefore defined Pfirrmann’s grade 1 NP tissues as healthy. Grades 2 and 3 NP tissues were excluded as controls because of their differentiation into fibroblastic chondrocyte phenotype. In the present study, NP cells exhibited completely distinct phenotypes according to culture conditions. NP cell proliferation was significantly limited in agarose hydrogel scaffolds compared with monolayer cultures. This result supports previous findings that culture in 3D agarose cultures prevents serial expansion of NP cells and differentiation into the fibroblastic phenotype [13]–[15]. NP cells share a common lineage with articular chondrocytes, with both cell types expressing the key chondrocyte genes *Col2A1* and *ACAN*; the expression levels of these genes are related to degeneration [21]. A microarray study found that expression of *A2M* and *CK18* in chondrodystrophic NP cells was elevated compared with annulus fibrosus and articular cartilage [11]. Therefore, we selected these genes as specific markers of NP cells. However, contrary to our expectations, in 3D culture, synthesis of ECM components was altered after 5 days and expression levels of *Col2A1*, *ACAN*, and *CK18*, which determine the phenotype of NP cells [11], [15], were low compared with those in monolayer cultures. These results indicated that the environment that surrounds NP cells and promotes redifferentiation through the secretion of Col2A1, ACAN, and COMP was not constituted after 5 days. Thus, the culture environment promotes differentiation of NP cells even when cultured within 5 days from encapsulation in agarose hydrogels. In contrast, after 10 days, cells encapsulated in agarose hydrogels displayed similar morphological characteristics to native NP cells of grade 1 and expressed increased levels of*Col2A1* and *ACAN* mRNAs compared with monolayer cultures. Further, the expression levels of *Col2A1*, *ACAN*, *COMP*, and *CK18* increased at day 25 in 3D agarose cultures compared with monolayer cultures. The levels of *Col2A1*, *ACAN*, and *COMP* mRNA expression were similar to those of freshly isolated cells. The expression of key chondrocyte genes, *Sox5* and *Sox6*, is required for notochord extracellular matrix sheath formation, notochord cell survival, and formation of NP cells [22]. Moreover, *Sox9* is required for expression of *Col2A1*, *ACAN*, and production of sGAG in NP cells [23]. We show here that at day 10, the levels of *Sox5* and *Sox9* mRNAs were similar to those of freshly isolated cells. These results indicate that the phenotype of the native NP cells lost under culture conditions was regained. This is the first report describing *Sox5* expression in 3D-cultured NP cells. Moreover, in monolayers, even after 10 days of culture, NP cell populations exhibited a fibroblast-like cell shape and expressed high levels of *Col1A1* compared with 3D agarose cultures and native NP cells. At day 25, NP cells expressed higher levels of *Sox5* and *Sox9* compared with native NP cells. These results suggest that NP cells differentiated into fibroblastic cells in monolayer cultures, while 3D agarose cultures promoted the expression of *Col2A1* and *ACAN* through enhancement of *Sox5* and *Sox9* expression. Long-term 3D culture spanning 25 days promoted chondrodystrophic NP cell redifferentiation through the reconstruction of the pericellular microenvironment, thus reconstituting the native tissue phenotype. Moreover, sGAG secreted by encapsulated NP cells was significantly greater in agarose hydrogels than in monolayers, and was increased in a time-dependent manner. Several studies have characterized the phenotypic response of NP cells on different substrates. For example, porcine NP cells cultured as monolayers exhibit similar mRNA expression levels compared with alginate cultures, while cells in the transition zone are relatively sensitive to culture conditions [15]. However, bovine NP cells exhibit enhanced proteoglycan synthesis in alginate or collagen gels in contrast to cells in monolayers [3]. In the present study, NP cells of CDBs were phenotypically similar to NP cells in long-term 3D agarose culture at day 25. Taken together, the results of the present study suggest that 3D cultures of NP can mimic cells that populate either native, healthy, or degenerated NPs. Degenerated human disc tissue spontaneously secretes a number of proinflammatory mediators [24]–[30]. In the present study, similar results were obtained using degenerated canine disc tissue. The importance of these molecules in the pathophysiology of symptomatic disc degeneration is increasingly recognized. For example, increased amounts of matrix MMPs, nitric oxide, prostaglandin E2 (PGE2), and TNF-α are present in herniated lumbar discs [29]. LPS induces matrix degradation and markedly stimulates the production by bovine disc cells of several cytokines, including IL-1β, -6, and -10, [30]. The results of our cell culture experiments provide clear evidence that LPS can effectively induce increased levels of the major proinflammatory cytokine and MMP mRNAs, and in this respect, mimic degenerated NP tissues. Taken together, we show that 3D scaffolds mimic the native NP microenvironment in long-term cultures and serve to illustrate the potential of LPS for studying NP cell cultures. Our findings support a pivotal role for culture microenvironment on chondrodystrophic disc cell behavior and further suggest that the length of is an important factor in 3D scaffolds. Because the phenotype of NP cells of CDBs is similar to that of humans, these results also suggest that the same basic mechanism of accelerated degeneration functions in human NP tissue.
